# A content analysis of food advertising in Arab Gulf countries during Ramadan

**DOI:** 10.1093/heapro/daz116

**Published:** 2019-12-10

**Authors:** Zainab Alyousif, Nahlah Alkhunain, Wendy J Dahl, Anne E Mathews

**Affiliations:** 1 Food Science and Human Nutrition Department, University of Florida, Gainesville, FL, USA; 2 Department of Health and Rehabilitation Science, Princess Nora bint Abdul Rahman University, Riyadh, Saudi Arabia

**Keywords:** food advertising, Ramadan, Eastern Mediterranean Region, holiday weight gain

## Abstract

Research has explored the link between exposure to marketing of foods high in energy and low in nutrients, and obesity in Western countries. The prevalence of obesity in Arab Gulf countries is similar to that of Western countries, yet the influence of advertising and frequency of exposure to advertising of foods on subsequent food choices and health is largely unexplored. This project sought to examine the number and quality of foods advertised on television during Ramadan in Arab Gulf countries. Television programming (36 h total), 12 h each for three stations, Alwatan, Dubai and MBC, was recorded. Food and restaurant advertisements (ads) were classified, totaled and analysed for dietary healthfulness using the Model SSCg3d. Of the total ads aired (*n* = 1473), food and restaurant ads were the most common (20.4%). The ad type and frequency varied among channels with restaurant ads most common on Alwatan, drinks and soda ads on Dubai, and sweet snacks and desserts ads on MBC (*p* < 0.001). Channels also differed regarding the frequency of dairy food ads (*p* < 0.001). Most food ads promoted less healthy foods similar to marketing practices in other countries with high rates of obesity. Many ads promoted foods high in energy, saturated fat, sodium and added sugar. This work signals the need to further understand the relationship between advertising of nutrient-poor foods, food behaviours and obesity in Arab Gulf countries and how advertising regulations may address this public health issue.

## INTRODUCTION

Obesity is a worldwide problem. In 2014, the prevalence of obesity was estimated at more than one-half billion adults worldwide (WHO, 2016). Increasing rates of obesity intensifies the global population’s risk of obesity associated non-communicable diseases. Persuasive and frequent marketing of less healthy foods, commonly termed energy-dense, nutrient-poor (EDNP) foods, has been implicated as a contributor to poor food selectivity and subsequent excess weight gain in many developed countries, specifically in children and youth (Sonntag *et al*., 2015). Countries of the Eastern Mediterranean Region (EMR) have comparable rates of obesity to that of the UK, the USA and other developed countries (Musaiger, 2011), yet the potential relationship between marketing of EDNP foods, eating behaviours and obesity in EMR countries is less well understood.

There are many factors associated with the increasing prevalence of obesity in the EMR including changes in lifestyle, dietary habits, physical activity and the social and cultural environment (Musaiger, 2011). Lifestyle behaviours associated with observing Ramadan may be another factor. During Ramadan, most adults practice daily fasting including self-denial of foods, beverages and smoking during daylight hours. Eating is only allowed during the time period between sundown and dawn and is considered a time for social gatherings and family events. Daily and nightly routines change (BaHammam *et al*., 2010), such as eating *Suhur*, which is the last meal before fasting begins at sunrise as well as delays in the start of school and work. Moreover, sleeping patterns may be altered during Ramadan as many people stay awake most of the night (BaHammam, 2005) which increases the time of sitting and watching TV (Al-Hourani and Atoum, 2007). Popular TV programming after sundown encourages families and friends to gather together until the early morning hours (BaHammam *et al*., 2010).

Although altered food intake and eating patterns, specifically daytime fasting, during Ramadan has been shown to contribute to weight loss that is quickly regained (Hajek *et al*., 2012). A meta-analysis of studies conducted in different countries with participants of varying backgrounds and dietary habits, showed that small changes in body weight during Ramadan were reversed after Ramadan (Sadeghirad *et al*., 2014). In the EMR, few studies have included communities from Arab Gulf countries. In one such study of Saudi families during Ramadan, the majority of participants self-reported an increase in body weight and attributed their weight gain to increased consumption of foods high in fat and carbohydrates, as well as decreased physical activity (Bakhotmah, 2011). This weight gain may be of significant concern given that in Western societies, holiday weight gain is a major contributor to excess annual weight gain (Schoeller, 2014).

Food marketing to children is widely thought to contribute to an environment that promotes obesity, and thus, modifying the food environment is a logical step towards obesity prevention (Sonntag *et al*., 2015). Food marketing uses persuasive techniques (Vilaro *et al*., 2017) to influence preferences, purchases and consumption of foods that are often in contradiction to dietary guideline recommendations (Keller and Schulz, 2011). Although less research examining how advertising affects food-related behaviour in adults exists (Mills *et al*., 2013), advertising of foods and beverages, particularly during Ramadan, may be a contributing factor associated with consumption of EDNP foods and increasing obesity in the EMR. Ramadan is a particularly critical period to study as a prospective analyses of weight patterns from the USA clearly document weight gain during the traditional holiday period (Schoeller, 2014). This weight gain is not compensated for in the following months, resulting in a modest, but significant annual weight gain. Ramadan, being 30 days every year, may be a similar critical period contributing to annual weight gain among people of EMR countries. During Ramadan, 79.4% of Saudi families reported an increase in their overall food expenditures primarily due to social reasons, including celebrations, and invitations to gatherings with family and friends (Bakhotmah, 2011). As families break their daily fast each evening of Ramadan, persuasive TV advertising that occurs during these same hours may be particularly influential on food purchasing and consumption behaviours. The aim of this study was to evaluate the frequency and quality of food and beverage advertisements (ads) broadcast on television in Arab Gulf countries during Ramadan.

## MATERIALS 

### Study design

The present study was carried out during Ramadan (July 2014) and is an observational report of television advertising that occurred on major television networks in three Arab Gulf countries, which share a similar culture. The networks selected were the three most widely broadcast channels in the Arab Gulf countries; MBC (Saudi Arabia), the Dubai channel (United Arab Emirates) and Alwatan (Kuwait). The percentage of the total population that identifies as Muslim in Saudi Arabia, Kuwait, and UAE are 97.1, 86.4 and 76%, respectively (NationMaster, 2011). Due to the changes in sleeping patterns and TV programming schedules during Ramadan, all recordings were made between 7:00 p.m. and 1:00 a.m. Twelve hours of programming were recorded over two evenings from each of the three channels for a total of 36 h of programming.

### Television ad categorization

Televised ads were reviewed and classified into one of 16 major categories: food/restaurant, mobile service company, cars, TV show/movie/play, cosmetics, home/bath products, bank and financial services, health care, contracting and investment companies, clothes/jewellery/watches, tourism, charity and donations, public service announcements, magazines/newspapers, electronics, school, and other. Food-related ads were further evaluated for both type and healthfulness of the advertised product. Food-related categories included drinks/soda, restaurants, sweet snack/dessert, dairy and other.

### Food healthfulness categorization, model SSCg3d

The healthfulness of food and restaurant ads was determined using the validated Model SSCg3d (Scarborough *et al*., 2007), a ‘nutrient profile model’ developed for the UK Food Standards Agency used to rank healthiness of advertised food during TV programming (Rayner *et al*., 2005). The model scores foods to a maximum of 10 points per nutrient/food component, based on their content of ‘energy, saturated fat, non-milk extrinsic sugars (NMES), sodium, calcium, iron, n-3 polyunsaturated fatty acids, and fruit and vegetables’ (Rayner *et al*., 2005). To calculate the overall score, the following formula was used: overall score = (total points from A category) – (total points from C category). Total A is the total score of energy, saturated fat, NMES and sodium, whereas the total C is the total score of ‘calcium, iron, n-3 polyunsaturated fatty acids, and fruit and vegetables’ (Rayner *et al*., 2005). A food or drink is classified as ‘less healthy’ when it scores nine points or more and as ‘healthier’ when it scores two points or less’. The categorization and calculations were completed by the main author and reviewed by one of the co-authors. All nutrition information was obtained from the products’ labels.

### Statistical analysis

Descriptive statistics were used to describe the types of ads aired during the study period on each television channel. A *χ*^2^ test was used to detect statistically significant differences between each food category, advertisement hours on each channel and between the three channels.

## RESULTS

**Fig. 1:**
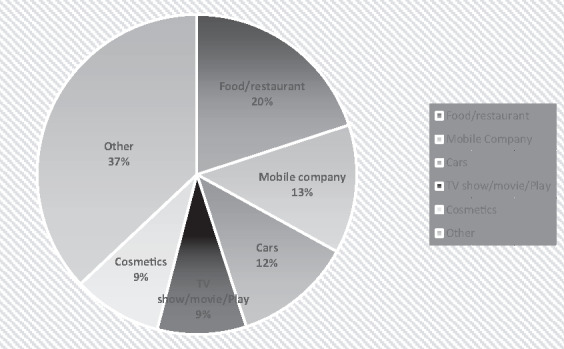
Advertisements, as a percentage of total, broadcast on MBC (Saudi Arabia), the Dubai channel (United Arab Emirates) and Alwatan (Kuwait) during Ramadan.

Across the 3 channels, there were a total of 1473 ads in 36 h of television programming during Ramadan. Overall, commercials promoting food/restaurants were the most frequently aired category and made up 20.4% of the total ads (Figure 1).

Food/restaurants was the top advertised category on Alwatan and Dubai channels and the third most advertised category on MBC, contributing 25.9, 17.3 and 15.8%, respectively. The number of food-related ads during each recorded hour was totalled (Table 1). The mean number of food/restaurant ads across the three channels was 8.3 ads/h, with Alwatan at 12.9 ads/h, Dubai at 6.6 ads/h and MBC with 5.4 ads/h. The most frequently advertised food groups were drinks/soda (42.6% of food ads), restaurants (22.3%), sweet snack/dessert (18.3%) and dairy (12.3%) (Table 2).

**Table 1:** Number of food-related ads on Gulf television stations during the evening hours of Ramadan, by hour and type

Food-related advertising groups		Time periods						
		7–8 p.m.	8–9 p.m.	9–10 p.m.	10–11 p.m.	11 p.m. to 12 a.m.	12–1 a.m.	*p* value
Drinks and soda	MBC	14	1	5	1	0	2	0.08
	Dubai	0	2	15	17	20	2	0.1
	Alwatan	6	9	0	8	13	13	<0.001
	Total	20	12	20	26	33	17	0.002
Restaurants	MBC	8	0	0	1	0	2	0.07
	Dubai	0	0	0	0	0	0	–
	Alwatan	21	6	3	3	6	16	<0.001
	Total	29	6	3	4	6	18	0
Sweet snack/dessert	MBC	1	5	7	2	6	5	<0.001
	Dubai	0	0	2	0	2	0	0.5
	Alwatan	5	8	12	0	1	0	<0.001
	Total	6	13	21	2	9	5	<0.001
Dairy	MBC	0	0	1	0	0	0	0.6
	Dubai	0	0	1	15	3	1	0.01
	Alwatan	0	6	10	0	0	0	<0.001
	Total	0	6	10	15	3	1	<0.001

Chi-square test was conducted to determine statistical significance. Statistical significance set at <0.05.

**Table 2:** The frequency and healthfulness of televised advertisements of foods and restaurants on Arab Gulf television stations during Ramadan

Advertisement categories	MBC ads/h	Dubai ads/h	Alwatan ads/h	Total, *n*	Percent of total (%)	SSCg3d category	*p* value
Drinks/soda	23^a^	56^b^	49^a^	128	42.7	55.5% less healthy 44.5% healthier	<0.001
Restaurants	11^a^	0	56^b^	67	22.3	N/A	0.004
Sweet snack/dessert	26^a^	4^b^	25^c^	55	18.3	100% less healthy	<0.001
Dairy	1^a^	20^b^	16^c^	37	12.3	97.3% less healthy 2.7% healthier	<0.001
Others	4	0	9	13	4.3	–	–
Number of food ads in 12 h	65	80	155	300	100	–	–
Total ads/h	34.3	38.5	49.9	–	–	–	–
Mean food ads/h	5.4	6.6	12.9	–	–	–	–

Chi-square test was used to detect statistically significant differences. *p* value < 0.05 is statistically significant. In each row: the difference between a, b and c are statistically significant.

ads, televised advertisements.

Drinks and soda products were the most frequently advertised food products on Dubai (70.0%) followed by MBC (35.4%) and Alwatan (31.6%). Model SSCg3d results showed that 55.5% of drinks/soda products were less healthy due to the high amount of sugar in each product while 44.5% of the advertised drinks were healthy (e.g. tea and water). Sweet snacks and desserts were the most frequently advertised foods on the MBC channel (40%) followed by Alwatan at 16.1% and Dubai at 5%. All sweet snack/dessert foods advertised during this period were less healthy according to Model SSCg3d due to energy, sugar and saturated fat. Restaurants was the second most advertised food category with 22.3% of the total food ads and was most frequently advertised on Alwatan (36.1%) versus 16.9% on MBC and 0% on Dubai. Only two dairy products were advertised during 36 h of Ramadan’s programming, a yogurt drink on MBC and a cheese on Dubai and Alwatan. Overall, 71.4% of foods advertised were classified as less healthy. The sweet snack/dessert category made up 53.3% of the total less healthy products while products from the drinks and soda category represented 33.3%. Moreover, dairy products and other food products represented 6.6% of the total less healthy food products.

Among the three channels, the distribution in frequency of ads from the food/restaurants category compared with other categories was significantly different (*p* < 0.001). Alwatan had more food/restaurants ads compared with other channels (*p* < 0.001); however, the difference between MBC and Dubai channels was not significant (*p *=* *0.5). Although the number of drinks/soda ads were significantly different between all channels (*p* < 0.001), the difference between MBC and Alwatan was not significant (*p* = 0.6). Restaurants, which were not advertised on the Dubai channel, were significantly higher on Alwatan than MBC (*p *=* *0.004). Sweet snack/dessert was also significantly different among all channels (*p* < 0.001). The Dubai channel had the lowest number of sweet snack/dessert ads compared with MBC and Alwatan. Channels also differed regarding the frequency of dairy food ads (*p* < 0.001). The number of food-related ads during each hour of the recording hours were reported (Table 1).

## DISCUSSION

This study showed that food and restaurant ads were the highest frequency (20.4%) of all ad categories. This is comparable to a Turkish study on advertising during evening programming that reported 29.7% of ads were food-related, averaging 5.6 ads/h of programming (Akcil Ok *et al*., 2016). In the present study, the Kuwaiti channel, Alwatan, broadcast 12.9 ads/h, exceeding the frequency of the evening food advertising on Turkish stations. A study conducted in 10 countries has shown that food advertising was high during children’s programming, ranging from 21 to 84% of ads, with the mean number of ads per hour ranging from 0.3 ads/h in Sweden to 11.5 ads/h in Australia (Lobstein and Dibb, 2005). However, in the present study, all evening programming was evaluated, not specifically children’s programming. Most of the food ads were for less healthy products. The percentage of healthier and less healthy products comparing to the total ads were 4 and 11%, respectively. In a similar study, which was conducted in the Islamic Republic of Iran, the percentage of less healthy EDNP products was 2.9%, which was less than our finding during Ramadan (Etemad *et al*., 2016).

As it has been demonstrated that weight gain may occur during Ramadan (Bakhotmah, 2011), the high rate of EDNP food advertising during this period may play a contributory role to overall obesity rates in the EMR. Saudi families who reported weight gain during Ramadan believed that they gained weight due to high intakes of fatty foods and excess carbohydrate rich foods (Bakhotmah, 2011). This eating pattern reflects the content of what we observed of most food products that were advertised during Ramadan programming. It is possible that weight gain following Ramadan is sustained, contributing to excess yearly weight gain, as has been shown with holiday weight gain in Western societies (Schoeller, 2014).

Alwatan aired the highest rate of food advertising as well as the most frequent restaurant ads, which were primarily for fast food and dessert stores. It is interesting to note that Kuwait has the highest prevalence of overweight/obesity of all Arab Gulf countries, totalling 74% of men and 77% of women (Ng *et al*., 2011). Kuwaiti families frequently have meals in restaurants and consume fast food (Al-Awadi and Amin, 1992). In the USA, fast-food restaurant advertising exposure has been shown to have a strong association with the probability of being overweight in children (Chou *et al*., 2008). It is possible that advertising of fast foods contributes to higher intake of these foods, excess energy and weight gain in Kuwait and other EMR populations. Sweet snack/dessert products ranked third for all channels and first for MBC as the most advertised food category. As expected, the advertised products were high in energy, saturated fat and added sugar. Prevalence of obesity in Saudi Arabia is second among the Arab Gulf countries with 28 and 44% of men and women, respectively (ALNohair, 2014). Women in some Arab countries, such as in Saudi Arabia, spend most of their time watching TV, socializing and snacking (Ng *et al*., 2011). Watching television increases food intake, including fast food, soda and high calorie snacks (Boulos *et al*., 2012), and the high rate of snack ads may further influence snacking behaviour.

In the present research, 8.7% of the total ads were related to beverages, unlike a study of ten selected Iranian TV and radio channels that showed only 0.7% of the total ads were related to beverages (Etemad *et al*., 2016). Frequent consumption of sugar-sweetened beverages is implicated in the rise in obesity worldwide (Massougbodji *et al*., 2014). Similar to reports from the USA, Australia and the UK (Lobstein and Dibb, 2005), drinks and soda products were among the most frequently advertised food products among all the three EMR channels. Of food ads on Dubai, which represents the United Arab Emirates, 70% were for fruit drinks and a local beverage popular during Ramadan. According to the Model SSCg3d, most of these products were less healthy due to the added sugar content. A study of Emirati women noted that two-thirds were overweight or obese, and excess energy consumption from beverages was suggested as a contributing factor (Ng *et al*., 2011). The consumption of beverages with added sugar was a significant contribution to overall energy intake in women and male children in the United Arab Emirates. Moreover, sugared sodas, such as cola and fruit drinks, represent 40–71% of liquid beverages consumed in the United Arab Emirates.

This study had limitations. In 2014, the World Cup soccer games coincided with Ramadan and many games were aired during the same evening hours. This may have influenced the type and frequency of television ads. Similarly, as this study was purposefully conducted during Ramadan, these results may not reflect the television advertising of food at other times of the year. While this content analysis of current television ads cannot conclude that food choice behaviours are being directly influenced, supporting literature from other geographic regions signal a relationship, at least in children (Boyland and Whalen, 2015). This project is a critical first step that may lead to a larger evaluation of current practices and policies of food marketing and its relationship to wellness in the EMR.

## CONCLUSIONS

Foods advertised during Ramadan are in contrast to dietary recommendations and can be described as less healthy, typically high in sugar, saturated fat and energy. This suggests that rampant marketing of EDNP foods is a concern not limited to Western countries but may be a more global public health concern. There is an emerging body of literature supporting the influence of marketing on food choice and that EMR countries are experiencing these same influences as western countries. Additional research is recommended to determine if exposure of adults and children to food advertising in the EMR is associated with weight gain during Ramadan, and if so, whether this weight gain contributes to excess yearly weight gain and obesity. 

## FUNDING

This project was supported by the Food Science and Human Nutrition Department, University of Florida.
